# The impact of Philosophy for Children (P4C) activities on enhancing the speaking skills of gifted students

**DOI:** 10.3389/fpsyg.2024.1451532

**Published:** 2024-10-22

**Authors:** Emine Balcı, Ramazan Eryılmaz

**Affiliations:** ^1^Educationa Faculty, Alanya Alaaddin Keykubat University, Antalya, Türkiye; ^2^Alanya Alaaddin Keykubat University, Antalya, Türkiye

**Keywords:** P4C, gifted students, speaking, skill, anxiety

## Abstract

The aim of this study was to investigate the impact of Philosophy for Children (P4C) activities integrated into Turkish lessons on the speaking skills of gifted students. Both quantitative and qualitative data were collected through interviews and various scales, employing a mixed-method design. The results indicated that students had positive feelings about P4C, including appreciation, curiosity, surprise, excitement, self-confidence, and empathy. They believed that P4C enhanced their problem-solving, creativity, questioning, effective speaking, and collaboration skills. However, some students also expressed negative views, tough less frequently, citing issues such as nonsense, limited time, the necessity of prior knowledge, and lack of idea diversification. Quantitative data revealed that P4C activities significantly improved the speaking skills of gifted students and notably reduced their speaking anxiety. Additionally, sex and program variables showed no significant effect on speaking skills and speaking anxiety.

## Introduction

Thinking skills hold significant importance in the context of rapidly evolving knowledge, technology, and global interaction (Güneş, 2021). These skills include competencies such as critical thinking, creative thinking, and problem-solving. One of the key expectations of students in the 21st century is to develop these abilities. Critical thinking is defined as the capacity to analyse information and evaluate different perspectives (Facione, 2011), and Paul and Elder (2014) regard it as an essential skill for solving complex problems. Creative thinking, emphasized as the ability to generate new and valuable ideas, has been advocated by Robinson (2015) as a crucial element that should be prioritized within education systems. Furthermore, the complex challenges of the digital age require students to apply knowledge effectively in novel situations (Dede, 2010). Problem-solving skills go beyond accessing and utilizing information; they involve applying knowledge in complex, unfamiliar contexts. Dede (2010) argues that problem-solving is a fundamental ability in the digital and global era, and education systems must prepare students accordingly. Acquiring these skills enables students to become effective individuals in the uncertain and complex world of the 21st century. Based on this, it becomes evident that it is crucial for students in the information age to have the ability to justify knowledge logically in terms of recognizing, defining, acquiring, and producing it. An approach that has garnered interest in the field of education worldwide, Philosophy for Children (P4C), offers practices that can serve this purpose (Tibaldeo, 2023; Lipman, 2023). The P4C approach is defined by Lipman (2023) as an educational method involving Socratic inquiries conducted with children. One of the fields related to the P4C approach is language education. In addition to developing students’ thinking skills, it appears that P4C also serves to enhance fundamental language skills (Lipman, 2023). In P4C, various solutions and opinions are developed through inquisitive, critical, and creative thinking processes in an environment conducive to discussion, using relevant concepts over a specific text, context, and problem (Lipman, 2023). Therefore, P4C activities appear to be conducive to conducting educational activities where fundamental language skills, particularly speaking, are used in higher-order cognitive processes and thinking styles (Özcan et al., 2023).

P4C can be a powerful tool for developing speaking skills, especially as it promotes critical, creative, and reflective thinking through dialog and inquiry. According to Lipman (2003), the Socratic questioning method used in a collaborative environment provides a space for students to articulate their thoughts in a structured and meaningful way. One of the most important factors in the development of speaking skills is the reduction of speech anxiety (Kankam and Boateng, 2017). In this context, the open and inclusive discussion-based approach of P4C can create a safe environment where students can express themselves without fear of judgment. Therefore, exploring the relationship between P4C and speech anxiety is crucial for understanding how philosophical inquiry can enhance not only cognitive skills but also core language competencies. Some children can exhibit rapid development compared to others in terms of attributes such as comprehension speed, thinking style, creativity, intuition, and imagination. These students are referred to as gifted students (Renzulli, 2002; Sak, 2013). In the field of education, special works are made for these gifted students. Therefore, enriched educational programs are needed to meet the specific needs of these students. On the other hand, identifying and educating individuals with special talent or gift is of strategic importance for a country. Science and Art Centres (SAC) in Türkiye are centres where students identified as gifted receive education outside of regular school hours (Millî Eğitim Bakanlığı, 2022). SACs aim to support, rather than replace, formal education (Delibay, 2017). In the 2022–2023 academic year, 67,375 students received special education in 353 SACs across Türkiye (Ministry of National Education, 2022).

Skills such as analysis, evaluation, questioning, transformation, comparison, matching, establishing cause-and-effect relationships, designing, and discussion are fundamental (Özmutlu and Uysal, 2021, pp. 522–526; Haladyna, 1997). Higher-order thinking skills, which are more advanced and involve these fundamental skills, include critical thinking, reflective thinking, creative thinking, and analytical thinking (Çevik, 2021; Yıldırım and Benzer, 2023; Olukçu and Yıldız, 2022; Uysal, 2022). P4C approach, which has gained considerable attention in recent years, is particularly intriguing for thinking education for students. Lipman and Sharp developed this approach and created the P4C program in the late 1970s (Tibaldeo, 2023). The program aims to develop students’ thinking abilities. Lipman and Sharp conducted an experimental study with stories suitable for this approach and obtained positive results (Tibaldeo, 2023). Later, Lipman and Sharp, prepared a handbook for teachers that includes many philosophical exercises (Lipman, 2003). Although there are arguments that the P4C approach is excessively mechanical in its evaluation of events and phenomena and neglects social values (Figueiredo, 2022), P4C remains of interest in language and thinking education. P4C involves providing stimulating questions to children, forming questions, creating a discussion environment, conducting discussions, and finally, steps of the discussion (Avcı, 2023). The pedagogical use of philosophy has gained strength since the 1970s. UNESCO, which aims to support education, science, and culture globally, has various initiatives in this area (UNESCO, 2007; Tepe, 2023). Speaking activities suitable for this approach may reduce students’ speaking anxiety. The level of speaking anxiety can also be explanatory in understanding students’ discussion culture and confidence levels. A student’s ability to comfortably express their opinions in a classroom or any community setting is related to individual or social conditions. Individually, the student must have linguistic skills to express their opinions and experience and preparation for public speaking. In this context, the teacher acts as a facilitator (Avcı, 2023). In P4C studies, researchers frequently focus on logical reasoning, reading comprehension, mathematical skills, listening skills, expressive language, creative thinking, critical thinking, and inquisitive thinking (Trickey and Topping, 2004; Canuto, 2018) as well as social areas like teamwork, flexibility, and empathy (Siddiqui et al., 2019; Jirásek and Jágerová, 2024). The use of narratives in philosophical inquiries has shown functional results (Stanley, 2007; Chamberlain, 1993; Canbaz and Kaplan, 2022; Çayır, 2021). In Türkiye, activities for children and youth in the P4C align with this understanding (Direk, 2018; Direk, 2011; Yılmaz and Bilican, 2022).

This study aims to reveal the impact of P4C activities implemented in BILSEM on students’ speaking skills and speaking anxiety and to identify students’ opinions on these activities. Various research questions have been determined for more detailed findings:

1. What are the opinions of gifted students regarding the P4C?
2. What is the effect of P4C on the speaking skills success scores of gifted students?
3. What is the effect of P4C on the speaking anxiety of gifted students?
4. Does the success in speaking skills after P4C activities differ by sex and participant’s program?

## Materials and methods

### Research design

Philosophical inquiry activities are aligned with a pedagogical approach that aims to develop individuals’ deep thinking, inquiry, and critical thinking skills. These practices encourage students to understand abstract concepts through discussion and to defend their own thoughts. P4C, developed by Lipman (2003) and Lipman et al. (1978), is considered one of the most popular implementations of this approach. In this study, philosophical inquiry activities were conducted with gifted students over 5 weeks, comprising 10 lessons. The effects of this process on students’ speaking skills and anxiety, as well as their opinions on the activities, were determined. Quantitative research approaches were used to measure speaking skills and anxiety, while qualitative approaches were used to determine students’ opinions. The embedded experimental design, one of the mixed methods designs that combines experimental research findings with participants’ perspectives, was chosen (Creswell and Clark, 2011). In this study, qualitative data were embedded within the quantitative data, with a greater emphasis on the quantitative data.

The study’s quantitative data were obtained through the implementation of P4C in accordance with the experimental research procedure (Büyüköztürk et al., 2023). The Speech Anxiety (SA) scale (Gündüz and Demir, 2021) and the Speech Observation Form (SOF) (Bozkurt and Akkök, 2019) were administered to the study group before and after the P4C activities. The SA and SOF were applied as pre-tests and post-tests, with this experimental procedure conducted on a single group. Therefore, the study employed a one-group pretest-post-test design, considered one of the weak experimental designs (Büyüköztürk et al., 2023, pp. 208–209).

The qualitative data of the study were obtained from participants’ views on the P4C activities. Consequently, the research, in which the researcher integrates qualitative and quantitative data sets to understand the research questions, is a mixed-methods approach (Creswell, 2021, pp. 2–8). In this study, an explanatory sequential design, one of the types of mixed methods research, was preferred (Creswell, 2021, p. 39).

### Participants and implementation

The study group consists of students diagnosed as gifted, who are receiving education at Batman BILSEM. Unlike regular classrooms, students in BILSEM are grouped according to program levels. In this context, the study group comprises students from the Support Education (SE, 4th grade), Individual Talent Recognition (ITR, 5th and 6th grades), and Special Talent Development (STD, 7th and 8th grades) programs. For the selection of participants, typical purposive sampling, one of the non-random sampling methods, was preferred. In this sampling method, a typical case that exhibits average characteristics is selected from many situations in the universe (Büyüköztürk et al., 2023, pp. 94–95). The target number of participants before the implementation was 82. During the implementation process, some participants were excluded from the study group based on their attendance in the lessons. The criterion was sufficient participation in the activities, ensuring a minimum of 80% attendance. According to these criteria, 22 participants were excluded, and the study group consisted of a total of 60 students (Table 1).

**Table 1:** Study group descriptive statistics.

			*n*	%
Quantitative	Sex	Female	28	46
		Male	32	54
	Program	SDT	22	36
		ITR	18	30
		SE	20	34
	Total		60	100
Qualitative	Sex	Female	8	40
		Male	12	60
	Program	SDT	8	40
		ITR	7	35
		SE	5	25
	Total		20	100

To plan the implementation process, the principles and methods of the P4C approach in the relevant literature were identified (Direk, 2018; Direk, 2011; Lipman, 2023; Tepe, 2023; Bowell and Kemp, 2022; Worley, 2010; Tibaldeo, 2023). Accordingly, the facilitator role, rather than that of a teacher, was adopted by the practitioner (Fisher, 2022; Çayır, 2021; Avcı, 2023). It was ensured that the inquiry groups maintained a democratic, pluralistic, and participatory climate and that the activities were conducted in accordance with the P4C approach. First, philosophical texts relevant to the discussion themes were selected and used to foster students’ conceptual thinking skills. Facilitator notes were prepared to guide the discussion process and sustain engagement.

This inquiry plan was implemented over 10 sessions across 5 weeks. Each session was structured to observe and assess students’ philosophical thinking processes and their development. Information regarding the P4C activities implemented in the study is presented in Table 2.

**Table 2:** P4C Activities implementation process.

Week	Activity/Text	Step 1	Step 2
1	The Horizon Gazer	Read (Stage 1) Discussion	Read (Stage 2) Additional investigations
	The Ring of Gyges	Read Discussion	Alternative Situations Additional investigations
2	The Unhappy Prince	Read Discussion	Alternative situations Additional investigations
	New Sneakers	Read (Stage 1) Discussion	Read (Stage 2) Additional investigations
3	Courage	Read Discussion	Additional investigations
	Animals and Humans	Read Discussion	Additional investigations
4	4′33”	Watching Video Discussion	Additional investigations
	The Island Republic	Read (Stage 1) Discussion	Read (Stage 2) Additional Investigations
5	The Voluntary Prisoner	Read Discussion	Alternative situations Additional investigations
	Winnie the Pooh’s Cake	Read Discussion	Alternative situations Additional investigations

The implementation was carried out in two stages. In the first stage, the selected text was read together, and at certain key points, conceptual questions, questions designed to create cognitive dissonance, and counterexamples were presented. These questions were carefully selected to encourage students’ critical thinking skills. In the second stage, additional deepening inquiries were conducted following the text.

### Data collection tools

In this study, observation, interviews, and surveys were used as data collection techniques (Büyüköztürk et al., 2023). The data collection instruments used in this context are explained in the following sections.

#### Speech skills observation form (SOF)

This scale is designed for observers to rate students’ speaking performances. The form consists of the sub-dimensions of Fluency, Pronunciation, Content-Language Use, and Interaction-Presentation Strategies. The scale is a Likert-type instrument with 30 questions, each with 4 options. The validity and reliability of the scale have been established (Bozkurt and Akkök, 2019).

#### Speech anxiety scale (SA)

This scale aims to determine the speaking anxiety of secondary school students. The causes of anxiety include self-perceptions or negative feelings and thoughts related to speaking. This scale consists of 30 items in a 5-point Likert format. The validity and reliability of the scale have been established (Gündüz and Demir, 2021).

#### Semi-structured interview form (SIF)

Interviews aim to understand a participant’s perspective on a particular situation by delving into their inner world (Patton, 2014). In this study, the interviews aimed to determine the views of SE, ITR, and SDT students on P4C activities and related concepts. Interviews can be structured, unstructured, or semi-structured (Creswell, 2013). Kvale (1994) outlines seven stages of an interview: thematizing, designing, interviewing, transcribing, analysing, verifying, and reporting. In this study, semi-structured interviews were conducted in accordance with this procedure.

This form is comprising four sections with a total of ten questions.

1. Feelings and thoughts on P4C activities: This section aims to understand students’ general impressions and personal preferences regarding the activities. It investigates which activities were most and least favored and the reasons behind these preferences.
2. Perceptions of the discussion environment: This part evaluates students’ experiences within the discussion environment. It focuses on skills such as expressing opinions, providing examples, listening to counterarguments, and adhering to discussion norms, including distinguishing between different types of statements and avoiding inconsistencies.
3. Impact of discussions on personal development: This section explores the contributions of the discussions to students’ personal learning and development. It also addresses students’ feelings and thoughts about philosophical questions.
4. Suggestions for future philosophical activities: Finally, this part gathers students’ recommendations for future P4C activities to inform and enhance the development of these activities.

The reliability of the interview form was assessed through expert reviews. Feedback was obtained from two specialists in addition to the researcher, confirming that the form is both valid and reliable.

### Data analysis

The qualitative data of the research were analysed using content analysis. In this study, the analysis of qualitative data obtained from the SIF was based on the model proposed by Huberman and Miles (2016). This model consists of three stages: data reduction, data display, and conclusion drawing. In the first stage, preparations are made for the coding process by taking notes on the data set, summarizing the data, and breaking it down into simple relationship sets (Huberman and Miles, 2016). In line with this, in the first stage of this study, parts of the data set that were not related to the relevant topics and concepts were removed. In the second stage, the coding of qualitative data was carried out. A computer program, considered functional for the coding and organization of qualitative data, was used for the analysis. The data were coded by subject matter experts. The coding procedure followed the qualitative data analysis approaches proposed by Huberman and Miles (2016) and Merriam (2015). In the quantitative data analysis phase, the distribution of pre-test and post-test scores from the SOF and SA scale was checked using the Kolmogorov–Smirnov test (Table 3).

**Table 3:** Descriptive statistics for quantitative data.

			Kolmogorov–Smirnov			Skewness	Kurtosis
Scale		*n*	*X*	sd	*p*		
SA	Pre-test	60	2.75	0.50	0.47	0.12	1.06
	Post-test	60	1.55	0.20	0.00	1.80	4.03
SS	Pre-test	58	2.72	0.46	0.13	−0.24	−1.3
	Post-test	58	1.53	0.18	0.02	1.76	5.15

Skewness and kurtosis values within the range of ±2 are necessary conditions for normal distribution (Mangal, 2002; Tabachnick and Fidell, 2013). As seen in Table 4, when examining the results of the Kolmogorov–Smirnov test and skewness and kurtosis values, the pre-test data for SA and SOF, including all sub-dimensions, exhibit normal distribution. However, the post-test data for SA and SOF, specifically for Pronunciation, Fluency, and Content-Language Use sub-dimensions, do not follow normal distribution. In contrast, the post-test data for Interaction-Presentation Strategies show normal distribution. Therefore, non-parametric tests should be used for the analysis (Büyüköztürk, 2018, pp. 67–70; Tabachnick and Fidell, 2013; Mangal, 2002).

**Table 4:** SOF pre-test and post-test mean scores Wilcoxon signed ranks test.

	*n*	*W*	*r*	*Z*	*p*
Positive ranks	2	80	−0.80	−6.00	0.00
Negative ranks	56	1,631			
Ties	0	0			
Total	58	1,631			

From the non-parametric tests, the Wilcoxon Signed-Rank Test was used to compare pre-test and post-test scores for SA, as well as for Pronunciation, Fluency, and Content-Language Use sub-dimensions of SOF. For comparing the post-test data of SOF Interaction-Presentation Strategies with sex variable, the parametric test, *t*-Test, was employed to assess the difference between scores of related measurement groups. Kruskal-Wallis test was conducted to examine whether there are differences in mean scores of post-test SA and SOF among programs of gifted students. Furthermore, for evaluating the post-test data of SA concerning the sex variable, the Mann–Whitney U test, preferred among non-parametric tests, was utilized (Mangal, 2002).

Two software programs frequently used in the analysis of qualitative and quantitative data were utilized in the data analysis.

## Results

### Opinions of gifted students regarding the P4C

**Figure 1:**
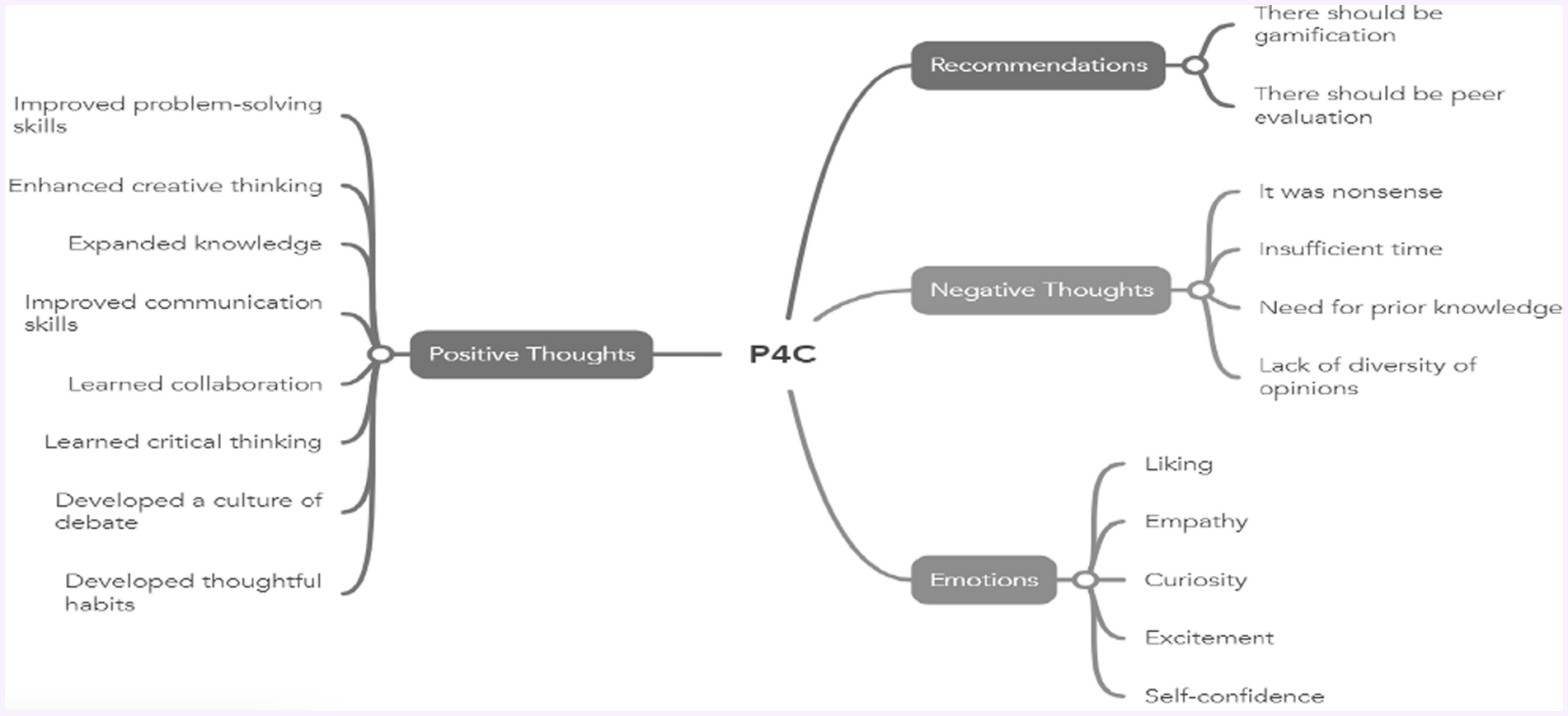
View on P4C.

Gifted students’ views, emotions, and thoughts regarding P4C have been categorized and coded. The relationships among themes, categories, and codes are depicted in Figure 1.

#### Emotions

In this category, five emotions have been coded: liking, empathy, curiosity, surprise, excitement, self-confidence. Most students expressed feelings of *liking* toward these activities (N:11, %55). *Self-confidence* was the second most reported emotion (N:8, %40). Additionally, students indicated that the activities stimulated their *curiosity (N:7, %35).* Furthermore, students showed interest in activities that involved *exciting* stories (N:5, %25). *Empathy* and *surprise were* also experienced by students at the same frequency (N:2, %10).

I enjoy philosophy activities and expressing my thoughts (S6 - Liking).

I developed greater respect for others and understood their feelings, recognizing that not everyone is the same (S1 - Empathy).

I aspire to be curious because I want to think like scientists and others who share this mindset (S17 - Curiosity).

Sometimes, there are surprises in the story that leave me feeling astonished (S12 - Surprise).

I liked the story of Gyges' ring because it was exciting, and I enjoy exciting narratives (S5 - Excitement).

I used to feel shy about expressing my thoughts, but now I no longer feel that way (S9 - Self-confidence).

#### Positive thoughts

Students’ positive perceptions of the Philosophy for Children (P4C) activities significantly outweigh their negative opinions. These favorable views are primarily related to the skills they acquire and the beneficial elements that impact their educational experiences. A notable number of students believe that these activities are helpful in *solving problems* they encounter during learning processes and assessments (N: 6, 30%). Another positive aspect identified is the development of *creative thinking* skills (N: 3, 15%). Many students associate P4C with the *expansion of their knowledge base* (N: 7, 35%). Furthermore, P4C is recognized for enhancing skills related to *self-expression* (N: 6, 30%). It promotes a culture of *collaboration and teamwork* (N: 4, 20%). Additionally, P4C contributes to the development of *critical thinking skills* among students (N: 4, 20%). The program also supports the cultivation of a *discussion culture* within the classroom (N: 8, 40%). Lastly, one of P4C’s contributions is fostering *careful thinking* habits (N: 5, 25%).

I believe that the activities have been very helpful to me; for example, thinking differently has made it easier for me to solve problems (S1 - It enhanced my *problem-solving* skills).

I now enjoy researching and exploring various ideas. Questioning is a valuable practice, as it leads to the discovery of new concepts (S12 - It fostered my *creative thinking*).

Standing firm on my curiosities is my right. For instance, I seek reliable information about things I do not know (S5 - My *knowledge base* has expanded).

These discussions have significantly contributed to my communication abilities. I used to feel shy speaking in small groups, but I now experience much less inhibition (S16 - My *expressive skills* have developed).

I found harmony with my friends as well. I truly enjoyed our collaborative efforts to gather information together (S4 - I learned to *cooperate*).

Initially, my thoughts may have seemed nonsensical or even incorrect; however, through reflection and discussion, I am able to arrive at more accurate conclusions (S6 - I learned to think *critically*).

My tolerance for differing opinions has increased (S10). It is rewarding for everyone to express their views and thoughts (S20 - My *discussion culture* has developed).

Now, before responding to questions, I take the time to think thoroughly and anticipate various outcomes, which has taught me to be more patient (S8 - My habit of *careful thinking* has improved).

#### Negative thoughts

While positive views on the Philosophy for Children (P4C) approach are predominant, there are also a small number of negative opinions expressed by students. Some students found certain philosophical inquiries to be *unmeaningful* (N: 2, 10%). Additionally, others mentioned that *time constraints limited* their ability to express all of their thoughts (N: 2, 10%). Some students indicated a *need for preparatory work* regarding the discussion content and expressed that they required prior knowledge (N: 3, 15%). The ability of students to generate original viewpoints and develop creative thinking skills is crucial within P4C. However, some students noted that there was a *lack of diversity in opinions* (N: 4, 20%).

Some aspects of the discussions sometimes seemed nonsensical to me (ö11). A few points felt meaningless (ö13 - *Unmeaningful*).

Occasionally, the discussions conclude, but at other times, not everyone gets to speak as much as they would like before the lesson ends (ö16 - *Insufficient time*).

If we engage in discussions related to what we have previously learned, it would help reinforce those concepts (ö3). I believe we should have reviewed certain topics before these activities (ö14 *- Prior knowledge is needed*).

Additionally, some classmates tend to echo my statements, which is not ideal (ö12 *- Lack of diversity in opinions*).

#### Recommendations

Some students hold positive views regarding P4C activities; however, they also believe that improvements are necessary in certain areas to enhance the effectiveness of these activities. Key suggestions include *the incorporation of gamification* (N:4, 20%) and *peer evaluation* (N:3, 15%). Some students expressed a desire for P4C activities to be designed in a game-like format. Additionally, there are students who feel that the individual opinions presented after philosophical inquiries, as well as the way these views are discussed, should also be evaluated.

My teacher, I believe it would be better if we incorporated game-like activities at the end (ö5 - *It should involve games*).

If there’s enough time, we might evaluate each other (ö9). I think it would also be useful to review what each person has said after the activities are completed (ö15 - *There should be peer evaluation*).

### P4C and speaking skills of gifted students

Data and statistical analyses regarding the impact of P4C activities on the speaking skills of gifted students are presented in Table 4.

According to Table 5, positive ranks (*n* = 56, +*w* = 1,631) clearly outnumber negative ranks (*n* = 2, −*w* = 80). Therefore, regarding the impact of P4C activities on speaking skills (*r* = −0.80), a significant and positive effect (*Z* = −6.00, *p* < 0.05) has been found.

**Table 5:** Wilcoxon signed ranks test in SOF discourse subscale.

	*n*	*W*	*r*	*Z*	*p*
Positive ranks	2	111	−0.81	−5.80	0.00
Negative ranks	56	1,599			
Ties	0	0			
Total	58	1,599			

The SOF consists of various sub-dimensions: pronunciation, fluency, content/language use, and interaction/presentation strategies. The aim was to examine the effect of P4C activities on these sub-dimensions of speaking skills by comparing pre-test and post-test mean scores. Table 5 shows the Wilcoxon signed-rank test for the pronunciation sub-dimension.

According to Table 5, positive ranks (*n* = 2, +*w* = 111) clearly outnumber negative ranks (*n* = 56, +*w* = 1,599). Considering the effect size (*r* = −0.80), it can be concluded that P4C activities have a significant and positive effect on pronunciation (*Z* = −5.80, *p* < 0.05).

All values are from positive ranks (*n* = 58, +*w* = 1711). Given the high effect size (*r* = −0.90) and other indicators, P4C activities show a significant and positive effect on fluency (*Z* = −6.60, *p* < 0.05) (Table 6).

**Table 6:** Wilcoxon signed ranks test in SOF fluency subscale.

	*n*	*W*	*r*	*Z*	*p*
Positive ranks	0	0	−0.90	−6.60	0.00
Negative ranks	58	1711			
Ties	0	0			
Total	58	1711			

Table 7 presents the Wilcoxon signed-rank test for the content/language use sub-dimension.

**Table 7:** Wilcoxon signed ranks test in SOF content/language use subscale.

	*n*	*W*	*r*	*Z*	*p*
Positive ranks	1	4	−0.95	−6.40	0.00
Negative ranks	57	1,536			
Ties	0	0			
Total	58	1,536			

According to Table 7, positive ranks (*n* = 1, +*w* = 4) clearly outnumber negative ranks (*n* = 57, +*w* = 1,536). With a high effect size (*r* = −0.95) and considering other indicators, P4C activities have a significant and positive effect on content/language use (*Z* = −6.40, *p* < 0.05).

Table 8 presents the *t*-test for the interaction/presentation strategies sub-dimension.

**Table 8:** SOF interaction/presentation strategies subscale *t*-test.

	*n*	*X*	Sd	*T*	*p*
Pre-test	58	2.59	0.42	−1.99	0.00
Post-test	58	1.48	0.18		

According to Table 8, the pre-test mean (*X* = 2.59) is significantly higher than the post-test means. This indicates a positive change in interaction/presentation strategies. Based on the *t*-test for pre-test and post-test values, P4C activities have a significant positive effect on the interaction/presentation strategies sub-dimension (*t* = −1.99, *p* < 0.05).

### P4C and speaking anxiety of gifted students

To examine the effect of P4C activities on speaking anxiety, pre-test and post-test scores were compared using the Wilcoxon signed-rank test.

According to Table 9, all values are from positive ranks (*n* = 56, +*w* = 1,599), indicating a decrease in speaking anxiety across all items. With a high effect size (*r* = −0.80) and other indicators (*Z* = −6.72, *p* = 0.00), it can be concluded that P4C activities have a significant and positive effect on reducing speaking anxiety (*Z* = −5.80, *p* < 0.05).

**Table 9:** SA scale pre-test and post-test mean scores Wilcoxon signed-rank test.

	*n*	*W*	*r*	*Z*	*p*
Positive ranks	0	0	−0.93	−6.72	0.00
Negative ranks	60	1830			
Ties	0	0			
Total	60	1830			

### In terms of program and sex variables, the impact of P4C on speaking skills and anxiety

Statistical analysis regarding the differentiation of speaking skills by program type is presented in Table 10.

**Table 10:** Kruskal-Wallis test of SOF post-test.

Program	N	Kurtosis	Skewness	*X*	Sd	*t*	*p*
SE	16	2.70	9.16	1.53	2.23	451	
ITR	18	0.54	−0.20	1.54	0.20	537	0.93
SDT	24	2.35	1.44	1.52	0.13	723	
Total	58	1.76	5.15	1.48	0.18		

Examining Table 10, the mean scores for SE 2 (*X* = 1.53), ITR 1 (*X* = 1.54), and SDT (*X* = 1.52) are very close, indicating no significant difference in scores among these program types (*p* < 0.05).

The difference in SOF scores by sex was tested using a *t*-test.

According to Table 11, the mean scores for female students (*X* = 1.53) and male students (*X* = 1.52) are nearly the same. Considering these values, there is no significant difference in scores based on sex (*p* < 0.05).

**Table 11:** SOF Post-test sex variable *t*-test.

Sex	*n*	Kurtosis	Skewness	*X*	Sd	*t*	*p*
Female	26	−0.26	−1.85	1.53	0.22	0.58	0.83
Male	32	1.52	1.82	1.52	0.15		
Total	58	1.48	1.82	1.48	0.18		

The Kruskal-Wallis test was used to examine the differentiation of speech anxiety in terms of the level/program the student attended.

When Table 12 is analysed, it is seen that the mean scores of SE (*X* = 1.57), ITR (*X* = 1.53) and SDT (*X* = 1.54) are very close. Therefore, it can be said that attending certain levels/programs does not create a significant difference in terms of speaking anxiety (*H* = 0.03; *p* < 0.05).

**Table 12:** Kruskal-Wallis test of SA post-test.

Program	*n*	Kurtosis	Skewness	*X*	sd	*H*	*p*
SE	18	4.27	1.99	1.57	0.25	0.03	0.98
ITR	18	0.16	0.39	1.53	0.18		
SDT	24	5.06	2.28	1.54	0.18		
Total	60	4.03	1.80	1.55	0.20		

Whether speaking anxiety differs in terms of sex variable was tested with Mann–Whitney U, one of the nonparametric tests, since the relevant data were not normally distributed (Table 13).

**Table 13:** SA post-test sex variable Mann Whitney u test.

Sex	*n*	*X*	*sd*	*Z*	*U*	*p*
Female	26	1.53	0.23	0.56	820	0.28
Male	34	1.56	0.18			
Total	60	1.55	0.20			

Accordingly, the mean scores of female students (*X* = 1.53) and male students (*X* = 1.56) are almost the same. Considering the related values, it is seen that there is no significant difference between sex and speaking anxiety post-test scores (*Z* = 0.56; *U* = 820; *p* < 0.05).

## Discussion

Participant/students have positive feelings toward P4C such as liking, curiosity, surprise, excitement, self-confidence, and empathy. Asgari et al. (2023) concluded that while P4C does not significantly impact in-class collaboration and altruism, it does make significant positive differences in empathy building and learning autonomy, and overall enhances social and emotional competencies. P4C is noted to enhance empathy, self-confidence, and awareness. Similarly, Diver (2022) found that integrating P4C into school curricula shifts competitive tendencies among students toward collaborative learning, and contributes to the development of ethical values, democracy consciousness, and critical thinking skills (Parkin, 2022). Primary school students with special talents found P4C beneficial for teaching philosophy discussion and respect for different ideas, expressing a continuous desire to engage due to its enjoyable nature (Chamberlain, 1993). This study identifies students’ feelings and thoughts toward P4C, including liking, empathy, curiosity, surprise, excitement, and self-confidence.

Participant/students hold positive views on P4C’s enhancement of problem-solving, creative, questioning, careful thinking, effective speaking, and collaboration skills. Chamberlain (1993) believes P4C enhances critical thinking skills among gifted students. Yurtbakan (2023) examined P4C’s impact on critical thinking skills and values education among gifted primary school students, finding it enhances critical thinking skills but does not significantly contribute to values education. Özcan (2022) determined that P4C-based questioning instruction enhances questioning, critical thinking, creative thinking, collaborative learning, and reflective problem-solving skills among both gifted and typically developing students. Another study by Özcan et al. (2023) concluded that P4C improves 5th graders’ listening and reading comprehension skills. According to the literature and the findings of this study, P4C can help gifted students develop higher-order thinking skills.

Participant/students have relatively infrequent negative views toward P4C, such as it being nonsensical, time-restrictive, requiring prior knowledge, and lacking diversified opinions. Additionally, they recommend incorporating peer assessment and gamification in P4C activities. Today, the use of digital technologies in language learning is a fundamental research area (Bal and Udül, 2021). Reviewing technological opportunities in the context of P4C could lead to more effective outcomes. Çayır (2015) highlighted negative aspects of P4C activities including time constraints, focus on outcomes rather than processes, investigations turning into competitions, occasional student boredom, inconsistencies, and overgeneralizations. Steel (2011) discusses P4C within the tradition of philosophy emphasizing wisdom and morality from ancient and medieval times (Aristotle, 2020), critiquing P4C for adopting a negative approach in wisdom education due to its focus on thinking skills and the utilitarian skill development approach brought about by globalization. According to related studies and the findings of this study, P4C requires a longer duration for effective implementation.

P4C activities conducted with gifted students have facilitated the development of their speaking skills. Similarly, positive effects of this approach are evident across all dimensions of speaking skills - articulation, fluency, content/language use, interaction/presentation strategies.

P4C activities significantly reduced speaking anxiety among gifted students. Research directly addressing speaking skills and speaking anxiety in the context of P4C is limited. However, there are indications that P4C enhances students’ reading and listening comprehension skills (Özcan, 2022; Özcan et al., 2023), and there are results suggesting it reduces speaking anxiety (Yurtbakan, 2023; Çayır, 2015; Çayır, 2021; Acar and Arslan, 2023; Asgari et al., 2023). Similar results were obtained in this study. Using P4C in language classes has the potential to enhance flexible thinking, questioning, active learning, collaboration, and inclusiveness.

No relationship was found between the sex or educational level/program of gifted students and their speaking skills or speaking anxiety. To date, there have been no studies examining P4C in terms of sex and grade level. This study concludes that these variables do not create a significant difference.

Should the gender variable be examined? The impact of gender on learning processes is a widely debated topic in educational sciences. Sadker and Sadker (1994) highlighted in their research that male and female students respond differently to classroom interactions and teaching methods, and these differences can influence learning processes. It has been observed that in discussion-based teaching methods, such as P4C, male and female students may exhibit different learning styles and levels of participation. Hyde (2005) also noted that gender differences play a significant role in learning and cognitive development, emphasizing the influence of gender on the development of emotional and social skills. This study has found that P4C has the potential to enhance skills such as empathy, collaboration, and critical thinking. Therefore, examining the gender variable could contribute to a better understanding of its effects on students’ value systems and help make educational policies more inclusive.

Based on the research findings, several recommendations can be made:

Supporting positive emotions: it can be beneficial to enhance positive emotions among students. Given the positive feelings (such as enjoyment, curiosity, and excitement) that gifted students have toward P4C, it is crucial for teachers to plan activities that foster these emotions during P4C activities. The ability of P4C to evoke feelings can help create more interactive and enjoyable discussion environments.

Contributing to the development of critical thinking and problem-solving skills: P4C has been found to enhance critical thinking and problem-solving skills. In this context, educational programs should include more P4C-based activities. Group projects should be encouraged to boost students’ creative thinking and collaboration skills.

Functional in reducing speaking anxiety: considering P4C’s potential to reduce speaking anxiety, it is recommended to use P4C in various contexts to help students who experience this issue overcome their anxiety.

All these recommendations can facilitate the effective implementation of P4C practices and contribute to the development of students. In future studies, it is recommended to highlight the various effects of P4C through empirical research.

## Data Availability

The original contributions presented in the study are included in the article/supplementary material, further inquiries can be directed to the corresponding author/s.
